# Balancing High Densities and Conservation Targets to Optimise Koala Management Strategies

**DOI:** 10.1002/ece3.72470

**Published:** 2026-01-12

**Authors:** Frédérik Saltré, Katharina J. Peters, Daniel J. Rogers, Joël Chadoeuf, Vera Weisbecker, Corey J. A. Bradshaw

**Affiliations:** ^1^ Biogeography Ecology & Modelling|Ngura Nandamari, School of Life Sciences University Technology Sydney Sydney New South Wales Australia; ^2^ Australian Museum Research Institute Sydney New South Wales Australia; ^3^ Global Ecology Partuyarta Ngadluku Wardli Kuu, College of Science and Engineering Flinders University Adelaide South Australia Australia; ^4^ Australian Research Council Centre of Excellence for Australian Biodiversity and Heritage EpicAustralia.Org.Au Wollongong New South Wales Australia; ^5^ Marine Vertebrate Ecology Lab, Environmental Futures, School of Science University of Wollongong Wollongong New South Wales Australia; ^6^ Department for Environment and Water Adelaide South Australia Australia; ^7^ School of Biological Sciences University of Adelaide Adelaide South Australia Australia; ^8^ Ur 1052 French National Institute for Agricultural Research (INRA) France; ^9^ Bones & Biodiversity Lab College of Science and Engineering, Flinders University Adelaide South Australia Australia

**Keywords:** fertility control scenarios, inhomogeneous Poisson process, matrix population model, spatial prioritisation

## Abstract

Conservation management becomes complicated when globally threatened species reach high densities locally, exceeding the carrying capacity of the ecosystem and causing damage. Managing high‐profile native species is particularly challenging, because ethical debates and public opposition to traditional control methods often prompt shifts toward strategies that prevent environmental harm rather than reducing populations. The koala ( *Phascolarctos cinereus* ) in South Australia exemplifies these challenges because, although it can damage the vegetation from high browsing pressure, culling is avoided due to public resistance. Therefore, managers have to consider costly and logistically constrained alternatives such as fertility control and translocation. Demographic models are valuable tools for predicting population dynamics, but their effectiveness depends on reliable population density estimates, often biased by expert‐elicited and citizen‐science data. We combined a point‐process model, an ensemble species distribution model, and a demographic model to project koala populations in the Mount Lofty Ranges over the next 25 years to assess the efficiency and cost‐effectiveness of fertility‐control interventions while accounting for sampling biases, habitat suitability, and local densities. We tested two hypotheses: (1) koala distribution is driven by rainfall, temperature, and soil acidity, with summer rainfall boosting habitat suitability, and (2) spatially targeted fertility interventions in high‐suitability areas are more cost‐effective than generalised strategies due to subpopulation connectivity. Our models confirmed that these three environmental factors shape koala distribution and that, in the absence of intervention, the koala population could increase by ~17‐25% in 25 years. Fertility control focusing on adult females emerged as the most cost‐effective (~AU$34 million) strategy, although it was slower at reducing population size compared to an intervention also sterilising female back young. While the choice of sterilisation scenario has minimal impact on overall costs, ethical considerations and long‐term conservation goals such as population density thresholds will have more influence on managing expenses effectively.

## Graphical abstract

The koala population in South Australia's Mount Lofty Ranges is increasing, raising concerns about overbrowsing and the need for sustainable management. Using combined demographic, point‐process, and species distribution models, we projected koala populations over 25 years to evaluate fertility‐control strategies. Our findings highlight rainfall, temperature, and vegetation as key drivers of habitat suitability, with targeted fertility control for adult females emerging as the most cost‐effective intervention (~AU$34 million).

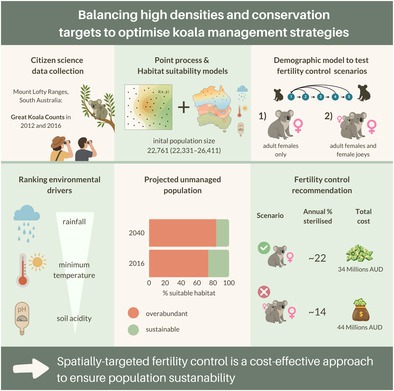

## 1 Introduction

Conservation management often faces conflicting objectives, particularly when species are listed as threatened at broader scales but exist at locally high densities (Woodroffe et al. 2014). When herbivores reach high densities, they can pose ecological challenges, including high grazing or browsing pressure that can alter vegetation structure and composition (Côté et al. 2004; Danell et al. 2006). Such impacts can degrade ecosystems, reduce their productivity (Van De Koppel and Rietkerk 2000) and associated faunal community, and ultimately decrease biodiversity (Foster et al. 2014).

Managing high‐density species presents different challenges depending on their inherent societal value (Drijfhout et al. 2020). While invasive species are often targeted for eradication (Coblentz 1990; Soulé 1990), controlling native species that are culturally significant or valued positively can trigger strong public resistance, making ecological decision‐making more complex. Although calls for population control (such as culling or translocation) are common when such species cause environmental or economic harm, control is controversial and often limited by cost, ecological side effects, and social acceptance (Dubois et al. 2017; Garrott et al. 1993). Given these challenges, conservation management of high‐profile species often favours alternative strategies that focus on preventing harm and restoring ecosystems rather than directly reducing animal numbers (Pressey et al. 2015).

Demographic modelling can contribute to the management of threatened species (McCarthy et al. 2003). Demographic models can guide population control by predicting population growth and long‐term viability under different climate and management scenarios (e.g., Jenouvrier et al. 2009), or by identifying the most effective strategies for managing high‐density or ecologically disruptive species (e.g., Govindarajulu et al. 2005; Venning et al. 2021). Despite uncertainties associated with model predictions, the approach remains a rigorous methodology that can use different types of data, incorporate uncertainties, and natural variabilities, to provide relevant predictions for conservation goals (Akçakaya and Sjögren‐Gulve 2000; McCarthy et al. 2003). However, these models require realistic estimates of initial population size (Caswell 2001), which, despite the development of many different field techniques designed for different species and survey conditions (Bookhout 1994), are still time‐consuming, costly, and logistically challenging to collect (Hauser et al. 2006).

Citizen‐science initiatives expand sampling effort (spatially and temporally), and provide new opportunities for cost‐efficient data collection (Silvertown 2009). However, they also present challenges, especially the risk of assuming that opportunistically collected data represent the true distribution and abundance of any species. Because species occupancy results from a hierarchical selection process (Johnson 1980), opportunistically collected data are conditional on observer presence and detection ability (Cretois et al. 2021). Volunteers frequently collect data opportunistically and subjectively (Fourcade et al. 2014), thereby introducing sampling biases in species distributions and abundance estimates (Crall et al. 2010). Moreover, observer skill in recognising the species and the time spent searching can vary widely, leading to a range of detection biases (Isaac et al. 2014) that can bias population estimates (Sicacha‐Parada et al. 2021), especially at fine spatial scales (e.g., 100s of metres). As a result, habitat selection estimates inferred exclusively from citizen‐science data only partially reflect true species distribution and abundance. Such estimates must therefore be corrected if they are to be used as reliable initial population sizes for demographic modelling.

The koala ( *Phascolarctos cinereus* ) exemplifies the tension between public perception and ecological reality, and therefore presents a compelling case study for applying demographic models supported by extensive citizen‐science data (Hollow et al. 2015; Sequeira et al. 2014) to manage conflicting conservation outcomes. As Australia's largest extant arboreal folivore and the sole surviving member of Phascolarctidae, the koala has a complex conservation history shaped by both biological and socio‐political factors. Although koalas are listed as ‘Endangered’ under the Commonwealth Environment Protection and Biodiversity Conservation Act (1999) in Queensland, New South Wales, and the Australian Capital Territory (EPBC 2023), and as ‘Vulnerable’ under the International Union for the Conservation of Nature (IUCN) Red List (iucnredlist.org), they receive no special conservation status in South Australia. Despite population declines in other regions of Australia due to habitat loss and climate extremes, koalas in South Australia (particularly in the Mount Lofty Ranges and Kangaroo Island) have reached high densities locally, creating ecological strain and management challenges. But despite causing environmental damage in some instances, South Australia's koalas are widely cherished by the public, making population control politically sensitive. Culling is largely avoided due to public opposition, prompting the use of alternative strategies such as fertility control and translocation. However, the associated costs, poor welfare outcomes, and logistical constraints have restricted these management options to small areas with a low potential for immigration (e.g., islands) (Massei and Dave 2014), where density targets have been arbitrarily set at ~0.70 koalas ha^−1^ (National Parks and Wildlife South Australia 2002; Ramsey et al. 2016). While proactive fertility‐control strategies in mainland regions could help to avoid drastic subsequent interventions (Whisson et al. 2016), the potential costs of these interventions in the Mount Lofty Ranges remain unknown.

We reconstructed and projected koala population dynamics across the Mount Lofty Ranges over the next 25 years to evaluate the relative cost‐effectiveness of possible fertility‐control interventions. By integrating population density estimates based on expert and citizen‐science data into habitat suitability and demographic models, we tested two hypotheses: (1) koala population distribution is driven primarily by a combination of rainfall, temperature, and soil acidity. We expect that increasing summer rainfall improves habitat suitability by mitigating the impact of rising temperatures, drought, and fire risks. However, extreme and low temperatures challenge the koala's ability to regulate body temperature, making access to suitable habitats—characterised by low soil pH—essential for survival. (2) Spatially targeted fertility control is expected to be more cost‐effective than broad‐scale strategies because it concentrates management efforts in areas where koala densities (and their potential ecological damage) are the highest. This targeted approach allows for more efficient use of resources and maximises the effectiveness of intervention (Baker 2017; Dorph et al. 2024; Pepin et al. 2017). We also anticipate that while some broad‐scale strategies might achieve faster reductions in population size, their higher implementation costs and logistical demands could ultimately render them less efficient in meeting long‐term management goals.

To test these hypotheses, we first overcame the aforementioned methodological limitations related to biases in citizen‐science data by developing an approach to estimate the initial, unbiased population size of the koala population in the Mount Lofty Ranges, and then constructed a demographic model to (i) project the effectiveness of various sterilisation intensities on the long‐term patterns of projected abundance, and (ii) estimate the costs associated with three sterilisation strategies: (1) no intervention, (2) only adult females sterilised, or (3) female back young and their mothers sterilised together at capture (see details in Methods). More specifically, we first developed an inhomogeneous Poisson process that accounted for the biases in uneven sampling effort in the two *Great Koala Counts* (i.e., citizen‐science initiatives aimed at monitoring the koala population in 2012 and 2016) that we coupled with a habitat‐based distribution model to estimate spatially averaged local densities across the Mount Lofty Ranges as a function of environmental conditions. We then used the resultant density estimates to calculate the initial unbiased population size in the demographic model to test the effectiveness of different sterilisation scenarios for reducing abundance relative to their associated costs. Ultimately, we identified the relative yearly costs of these sterilisation scenarios to provide the cheapest and most effective means of achieving population control over the next three decades.

## 2 Methods

### 2.1 Study Area

The Mount Lofty Ranges (35° S, 138.7° E) of South Australia are a region adjacent to the capital city of Adelaide, including the Adelaide Hills and Fleurieu Peninsula. The region receives 400–1100 mm rainfall annually within an otherwise semi‐arid landscape (Westphal et al. 2003). From its total area of 5000 km^2^, only ~10%–18% of native woodlands remain (Bradshaw 2012), with overstoreys dominated primarily by eucalypt species ( *Eucalyptus baxteri* , *E. fasciculosa*, *E. leucoxylon* , *E. obliqua,* and *E. viminalis* ). The rest of the region is devoted primarily to urban and peri‐urban residential housing, pasture, plantations, cropland, vineyards, and orchard agriculture (Bryan 2000; ForestrySA 2014). There are no records of koalas in this region during the Holocene (~12,000 years ago) prior to European invasion (Robinson et al. 1989); instead, the current population is derived from deliberate translocations from Kangaroo Island (Duka and Masters 2005; Melzer et al. 2000), as well as escaped animals from Cleland and Belair Wildlife Parks (Robinson and Bergin 1978).

### 2.2 Koala Distribution Data

Koala occurrence data were collected during two events of the *Great Koala Count* on 28 November 2012 and 26–27 November 2016, mainly in Adelaide and the Mount Lofty Ranges of South Australia (Hollow et al. 2015; Sbrocchi et al. 2015; Sequeira et al. 2014). As part of these surveys, citizen scientists were tasked with searching for koalas on the specified days of the surveys and reporting both sightings and non‐sightings (i.e., presences and absences). Reports could be made through the *Great Koala Count* website (koalacount.ala.org.au), or in near‐real time via Apple and Android smartphone apps adapted from existing mobile applications created to feed citizen‐science data to the *Atlas of Living Australia* (ala.org.au) (Stenhouse et al. 2020). Data collected included: (i) location (longitude and latitude, recorded by mobile GPS), (ii) a photograph for sighting validation, (iii) search effort in minutes, (iv) descriptions of the activity of the observed koala(s) (e.g., *sleeping*, *sitting*, *eating*, *climbing*, *drinking*, *walking*, *dead*, *other*), (v) whether participants expected to spot a koala in the area, (vi) location type (e.g., *private garden*, *public park*, *roadside*, *on road*, *other*), (vii) sighting frequency in the area, (viii) species of tree in which the koala was sighted, (ix) presence/absence of offspring, (x) tree health (e.g., *dead*, *lots of leaves*, *scarce leaves*, etc.), and (xi) any additional comments. We quality‐checked all records by removing duplicates (i.e., those with identical times, dates, and observers) or obviously erroneous entries (e.g., other species), resulting in a total of 1764 recorded sightings across the Mount Lofty Ranges (Table S1).

### 2.3 Sampling Bias Correction and Density Estimates

Citizen‐science data are inherently biased and the South Australian *Great Koala Count* datasets are no exception, with three main sources of bias identified (Sequeira et al. 2014). First, the data are strongly clustered around the frequently visited Cleland Wildlife Park, where a local peak in the density of koala detections describes a higher probability of detection the closer the observer is to the park. The second bias is also related to the presence of a national park (although the effect is not as pronounced as for Cleland) because observers appear more likely to detect a koala inside compared to outside a national park. Finally, the distance to the nearest road is also a strong driver of variation in sampling effort (Sequeira et al. 2014), so that the closer a koala is to a road, the higher its probability of being detected. This is likely due to increased observer access rather than the proximity of observers per se. These sampling biases must be corrected to produce reliable density estimates for input into our demographic model. We acknowledge additional sources of bias in the dataset such as koala sightings often clustered along walking trails within parks. It is also possible that some high‐density paths align with drainage features or creek lines, although we lacked access to fine‐resolution spatial data to explore this further. Regardless, citizen‐science datasets in this context should be treated as point patterns degraded by multiple factors, including uneven sampling effort, variation in detectability, and the potential for misidentification.

We described the spatial pattern of the censused koala population across the Mount Lofty Ranges using an inhomogeneous Poisson point‐process model assuming that (i) individual koalas do not have strong social interactions that could affect their spatial distribution (i.e., spatial locations are independent), (ii) koalas do not live in large groups, (iii) they are not aggressively territorial, and (iv) the probability of detecting a koala is conditional on local environmental conditions but independent of the probability of detecting another individual koala in the area. This point process can account for the distance an observer is from a koala‐detection hotspot (i.e., Cleland National Park), whether the observer is inside or outside a national park, or the distance an observer is from the nearest road.

The homogeneous Poisson process (N) is a suitable model when the points are ‘randomly’ (i.e., the location of each point does not depend on the location of its neighbours) distributed in space (Illian et al. 2008). This process is characterised by two fundamental properties: (i) the number of detections N in any subset of the study area C follows a Poisson distribution with mean $\mathrm{\lambda v}\left(C\right)$, where λ (intensity or point density) = the mean number of points per unit area, and v = a neutral symbol referring to the area (in km^2^), and (ii) the number of detections N in k disjoint subsets within $C_{k}$ generate k independent variables (for an arbitrary value of k).

In an inhomogeneous Poisson process, λ varies with location x on C, which in our case translates to a change in sighted koala density across the Mount Lofty Ranges, driven by the heterogeneous sampling effort of citizen scientists. By estimating $\lambda \left(x\right)$, we therefore obtain an average estimate of the density of the koala population across the Mount Lofty Ranges while accounting for biases in sampling effort and assuming that each koala has only been counted once. Based on these two fundamental properties, the probability of detection of a sighted koala in k non‐overlapping areas $C_{i}$ follows an inhomogeneous Poisson point process:

(1) $$P\left(N\left(C_{i}\right)=n_{i},i=1,\ldots ,k\right)=\prod_{i=1}^{k}\frac{{\left(⋀\left(C_{i}\right)\right)}^{n_{i}}}{n_{i}!}e^{-⋀\left(C_{i}\right)}$$

where $⋀\left(C\right)=\int_{C}\lambda \left(x\right)\;\mathrm{dx}<\infty$, and the intensity function $\lambda \left(x\right)$ can be estimated using a likelihood function:

(2) $$L\left(x_{i},i=1,\ldots ,k\right)=\lambda^{n}\frac{\prod p\left(x_{i}\right)}{n!}e^{-\lambda \;\int_{C}p\left(x\right)\;\mathrm{dx}}$$

where $p\left(x\right)$ = the probability of a koala detection at a given x location as a function of (i) the distance to the density hotspot (i.e., Cleland National Park), (ii) the probability of being inside or outside a national park, and (*iii*) the distance to the nearest road, such that:

(3) $$p\left(x\right)=p_{o}\left(x\right)e^{-\mathrm{ad}\left(x,h\right)}\;e^{-cd_{r}\left(x\right)}$$

where $e^{-cd_{r}\left(x\right)}$ represents the decreasing probability of a koala detection as a function of $d_{r}\left(x\right)$ = distance from x to the nearest road, and $e^{-\mathrm{ad}\left(x,h\right)}$ = decreasing probability of a koala detection as a function of $d\left(x,h\right)$, such that the greater the distance x is from a hotspot of detection h, the less likely it is to detect a koala. We also assumed that a koala cannot be missed at a short distance (e.g., < 10 m) from the observer and that the koala will not try to escape and avoid detection as the observer is approaching. The probability of detecting a koala inside and outside a national park is:

(4) $$p_{o}\left(x\right)=1_{\left\{x\in \text{park}\right\}}+b\times 1_{\left\{x\notin \text{park}\right\}}$$

with $1_{\left\{x\in \text{park}\right\}}$ being the indicative function that x is located inside a park (=1 if true, otherwise = 0) and $b\times 1_{\left\{x\notin \text{park}\right\}}$ the indicative function that x is located outside a park (=*b* if true, otherwise = 0). The parameters $\hat{a}$, $\hat{b}$ and $\hat{c}$ are estimated by maximum likelihood: here, $\hat{a}$ = 0.26, $\hat{b}$ = 0.18 and $\hat{c}$ = 7 × 10^−4^.

We calculated a confidence interval for $\hat{\lambda }$ using a parametric bootstrap approach (Manly 2006). We first simulated *n* = 1000 independent inhomogeneous Poisson processes of sighted koalas based on the estimated parameters $\hat{\lambda },$ $\hat{a}$, $\hat{b}$, and $\hat{c}$, such that each inhomogeneous Poisson process follows the same spatial pattern and characteristics as the dataset (i.e., *Great Koala Counts*). We then estimated for each of the $n_{i}$ simulated inhomogeneous Poisson processes the parameters $\left(\lambda \prime_{i},a\prime_{i},b\prime_{i},c\prime_{i}\right)$ based on Equations ((1), (2), (3)), which results in a vector of 1000 estimates per parameter. We subsequently calculated the confidence interval for each parameter ($\hat{\lambda \prime },$ $\hat{a\prime }$, $\hat{b\prime }$ and $\hat{c\prime }$) as the quantiles at 0.025 and 0.975 of the n values in each vector.

### 2.4 Species Distribution Model

#### 2.4.1 Model Overview

We used an ensemble of nine correlative species distribution models to estimate koala habitat suitability across the Mount Lofty Ranges as a function of nine environmental variables (see *Environmental Variables*). Correlative species distribution models predict and map species habitat suitability by estimating the statistical relationship between in situ occurrence (i.e., koala observations from the *Great Koala Counts*) and the environmental conditions of those locations. This statistical relationship is needed to capture the envelope of all suitable environmental conditions for a species to survive and thrive, which represents the realised environmental niche of the species (Guisan et al. 2017).

Among the broad range of available statistical algorithms to predict species distributions, we used an ensemble modelling approach based on nine widely used algorithms: artificial neural networks, generalised additive models, generalised linear models, boosted regression trees, flexible discriminant analysis, multivariate adaptive regression splines, maximum entropy, random forest, and species‐range envelopes. Each algorithm returns a map of suitable habitat for the species (i.e., nine in total) that generates a weighted‐mean consensus map (i.e., the relative contribution of each algorithm on the final map depends on its relative performance—see *Model Training, Performance, and Projections*). This ensemble approach integrates models of different complexities and statistical properties when projecting a species through time (Araújo and New 2007; Elith et al. 2011) and ensures that several possible projections are considered for mapping both the main trend (i.e., mean, median, or some other percentile) and the overall variation (and thus uncertainty) across all models (Figure S3). By combining different sources of information and algorithms, ensemble models can outperform single models, leading to more robust predictions under climate change scenarios (Araújo and New 2007; Forester et al. 2013).

Some of the algorithms we used in the ensemble modelling approach require either presence/absence or presence‐only data. We discarded the absence data collected in the *Great Koala Count* because of their lack of reliability. True absences are usually estimated based on repeated surveys and using multiple methods (Woosnam‐Merchez et al. 2012), which was not the case for the *Great Koala Counts* (e.g., most people only started their survey when they spotted their first koala). Therefore, we generated 2000 pseudo‐absence data by randomly sampling points for each species within the study area where the focal species was not recorded (Barbet‐Massin et al. 2012). Although the number of pseudo‐absences required can vary depending on the type of model, performance is generally highest with a large number of pseudo‐absences (e.g., 10,000) and/or with a 10:1 ratio of pseudo‐absences to presences (Barbet‐Massin et al. 2012; Guisan et al. 2017).

#### 2.4.2 Model Training, Performance, and Projections

We first randomly split our dataset (including pseudo‐absences) into 80% training and 20% validation subsets. To account for the stochasticity in pseudo‐absence generation, we repeated this process 20 times, thus generating 20 different training and evaluation datasets. We then computed each of the nine models independently and applied *k*‐fold cross‐validation (Fielding and Bell 1997) to evaluate performance using the 20% validation subset.

We evaluated model performance for each repetition using the area under the receiver operating characteristic curve (AUC) and the true skill statistic (TSS), two intuitive metrics to assess the predictive performance of species distribution models transposed into presence‐absence mapping (Allouche et al. 2006; Swets 1988). From the relative suitability map generated by each model for each repetition, we determined a threshold maximising TSS (which includes both sensitivity and specificity) (Guisan et al. 1998) below which we considered the species ‘absent’. This threshold method is commonly used to transform continuous probabilities of suitability into probabilities of presence/absence in species distribution models (Nenzen and Araújo 2011).

We projected to the complete study site and averaged predictions for each model across the 20 repetitions. We then generated the final ensemble projection averaging the predicted occurrences across all models, while weighting each model's contribution to the average based on its respective TSS (Thuiller et al. 2009), assuming that TSS is more reliable than AUC as a measure of accuracy when using dichotomous presence/absence data (Allouche et al. 2006). Models with higher TSS thus had a greater contribution to the ensemble estimate.

#### 2.4.3 Environmental Variables

Selecting spatially explicit environmental variables that approximate the species' niche based on its ecophysiological needs is an essential part of habitat suitability modelling (M. Austin 2007; M. P. Austin 2002; Mod et al. 2016). Ideally, these variables capture three primary ecological drivers (Guisan and Thuiller 2005; Guisan and Zimmermann 2000): (i) limiting factors that constrain metabolic processes, (ii) disturbances, either natural or anthropogenic, and (iii) resources (Guisan et al. 2017; Sequeira et al. 2014). Based on these principles, we selected the ensuing eleven environmental variables to build our species distribution models to predict koala habitat suitability: (1) minimum temperature (°C), (2) distance to water bodies (m), (3) average rainfall for November (mm), (4) total water index, (5) likelihood of native vegetation being present in the grid cell (%), (6) distance to roads (m), (7) solar exposure (megajoules m^−2^, MJ m^−2^), (8) water vapour pressure (in hectopascals, hPa), (9) elevation (m), (10) pH CaCl_2_ that reflects soil acidity (unitless), and (11) phosphorus content (% of fine soil mass). Variables such as water vapour pressure, solar exposure, distance to water bodies (defined as year‐round and seasonally inundated areas, a proxy for water availability), water index (a proxy for soil moisture) impose strong ecological, behavioural, and physiological constraints on koalas (Clifton et al. 2007; Ellis et al. 2010; Sequeira et al. 2014). Solar exposure and minimum temperatures are closely linked to heat stress that becomes high during dry periods with limited water access, especially for mammals that rely on evaporative cooling (Albright et al. 2010; Krockenberger et al. 2012). High koala mortality has been recorded during extreme heat events coinciding with low rainfall (Gordon et al. 1988), and individuals at the arid edge of their range experience increased physiological stress (Davies et al. 2013). Koalas are specialist marsupial folivores that can also select tree species based on their foliage water content, which is indirectly linked to temperature (Clifton et al. 2007). Koalas have a strong dietary preference for a few eucalypt species (Moore and Foley 2000; Tyndale‐Biscoe 2005) such as *Eucalyptus viminalis* (manna gum) and/or *E. ovata* (swamp gum) (Menkhorst 2008; Whisson et al. 2016). Koalas also prefer areas with higher soil and foliage phosphorus, which supports their nutritional needs and is linked to greater densities of preferred eucalypt species (McAlpine et al. 2023), and soil pH affects eucalypt biogeography by constraining nutrient availability (Bui et al. 2017; Ding et al. 2021). Topography influences microclimate, vegetation composition, and resource availability so that koalas predominantly favour slope aspects that offer more thermally favourable conditions (Mitchell et al. 2021). Distance to roads affects koala distribution estimates by (i) serving as a proxy for anthropogenic impact and (ii) introducing a detection bias in the point process model (distance is consistently identified as one of the strongest spatial predictors of koala sighting density) (Geldmann et al. 2016; Sequeira et al. 2014; Stenhouse et al. 2020).

We obtained spatial data on vegetation, topographic water features, transport infrastructure (distance to roads), and elevation from the Department of Environment and Water, Government of South Australia (data.sa.gov.au, Figure S1). We extracted soil pH and phosphorus content from the Soil and Landscape Grid of Australia (Malone and Searle 2024; Viscarra Rossel et al. 2014). We extracted climate data from the Australian Government Bureau of Meteorology (bom.gov.au), including 20‐year monthly averages (1993–2012) for minimum temperature, water vapour pressure, solar exposure (excluding November 2009, for which no data were available), and rainfall. We selected these variables with a focus on the month of November, aligning with the timing of the *Great Koala Count* surveys. Topography, total wind exposure, and native vegetation cover were available at an original spatial resolution of 1 arc sec (~30 m), and soil pH and phosphorus content were provided at 3 arc sec (~92 m). Variables such as distance to sealed roads, minimum temperature, distance to water, rainfall, solar exposure, and water vapour pressure were at a 1‐km^2^ resolution. To ensure consistency and align with the spatial scale of our species occurrence data, we resampled all environmental layers and projected them to a recommended uniform 1 km^2^ scale (Rhodes et al. 2009). We calculated the variance inflation factor for all climate variables and ensured that all variables returned a variance inflation factor < 10 to minimise multicollinearity.

#### 2.4.4 Variable Importance and Response Curves

We estimated the individual contribution of all variables in the species distribution models (Thuiller et al. 2009) for each of the nine statistical algorithms based on their present‐day projection as a benchmark. We then ran these algorithms with one environmental variable changed (randomly reshuffling that variable's values) while maintaining the others in the observed order. We then calculated Spearman's *ρ* between the new prediction and the benchmark prediction as a metric of relative variable importance (high *ρ* indicates that the randomised variable has little effect on final predictions). We repeated this process for each environmental variable in all 20 training datasets (10 iterations per variable). We subsequently calculated the mean and standard deviation of variable importance for each variable across the 10 iterations per algorithm, and then calculated the ensemble predictions using the TSS‐weighted average of the nine model algorithms.

We evaluated the responses of the species distributions to the gradients of explanatory variables based on the response curves derived from each model. We generated response curves by holding *k–1* variables constant at their mean value while the variable of interest contains 100 points varying from the maximum to the minimum of its range. Here, the variation in predictions for these 100 cells only reflects the effects of one selected variable. Thus, a plot of these predictions visualises the modelled response to the variable of interest, contingent on the other variables held constant.

#### 2.4.5 Abundance Estimates

With no specific information available for koala local abundances, we converted habitat suitability predictions from the ensemble species distribution model into local population densities using a quadratic relationship (VanDerWal et al. 2009). This function assumes that koala density peaks (corresponding to the average population density estimated by the inhomogeneous Poisson process) at intermediate suitability values and declines toward the extremes, reflecting ecological realities observed in the field. We calculated local densities for each cell and summed across the 3080 km^2^ Adelaide–Mount Lofty ranges study area to provide a total population estimate, with uncertainty bounds provided by the 95% confidence interval of the average population density estimated by the inhomogeneous Poisson process. This approach produced spatially explicit estimates of both habitat suitability and population size, directly supporting management and conservation planning.

### 2.5 Sterilisation Demographic Model

#### 2.5.1 Model Overview

We developed a 13 × 13 age‐classified (Leslie) matrix population model (i.e., a 12‐year age‐classified model produces a 13 × 13 matrix including the 0–1 year transition based on the longevity reported for koalas) (Smith 1979) to simulate the effect of fertility control for the koala population in the Mount Lofty Ranges. This is a female‐only model based on a sex ratio of 1:1 that is often used for population viability analyses because population growth is primarily influenced by females (Coulson et al. 2001), the reproductive potential of males is less limiting than that of females (Cope et al. 2018), and most large‐scale Australian koala fertility‐control projects have female‐focused efforts (e.g., Hynes et al. 2010; Ramsey et al. 2021; Watters et al. 2021). We therefore halved the total population size to estimate the initial number of females, in line with typical koala sex ratios (Ellis et al. 2010). As such, modelling females only still yields accurate projections of total population dynamics.

We gathered input data from previously published studies on wild koala populations across Australia. For fertilities, we acquired median values from Rhodes et al. (2011) (Figure S4), and for survival, we combined data from Penn et al. (2000), Dique et al. (2003), Lunney et al. (2007), and Rhodes et al. (2011) (Table S2). These fertility values likely represent or even potentially overestimate the true fertilities of koalas in the Mount Lofty Ranges. For example, ~85% of 268 adult females sampled between 2021 and 2024 were without young (not accounting for undetected pregnancies or recent mortality of joeys; Karen Burke da Silva, Flinders University, unpubl. data).

To combine the survival estimates across available studies, we developed a resampling approach where we first compiled the median and upper/lower limits of age‐specific survival per study (i.e., ±1.96 reported standard errors or confidence limits provided), and then standardised these uncertainties by back‐calculating a standard deviation for each class per study. From this dataset, we randomly resampled 10,000 medians and standard deviations per age class (interpolating missing data for a given age class from the mean of values for that age class), and then beta‐sampled age‐specific survival probabilities per iteration using the resampled medians and standard deviations. To smooth the stochastically resampled survivals, we applied an exponential association function:

(5) $$s_{i,x}=a_{i}e^{-e^{b_{i}-c_{i}x}}$$

where *s*_*i,x*_ = is the smoothed survival probability for age *x* in iteration *i*, and *a*_*i*_, *b*_*i*_, and *c*_*i*_ are constants per iteration *i*. From the 10,000 estimates of *a*, *b*, and *c*, we took the mean and upper and lower 95 percentiles per age *x* from which we resampled stochastically following a beta distribution in the matrix projections (Figure S2). Our model was female‐only, assuming a pre‐census design and a 1:1 sex ratio (Ellis et al. 2010; McLean 2007).

For each projection scenario (see *Projection Scenarios* and *Costs* sections), we stochastically resampled the age‐specific fertilities assuming a 3% standard deviation (Rhodes et al. 2011) and the survival probabilities from the smoothed mean values (±5% standard deviation, see Equation 5). We assumed a Gaussian distribution around the mean of fertility and a *β* distribution for survival probability (Table 1). We calculated the population's stable age distribution from the deterministic matrix (Caswell 2001), and then multiplied this stable age structure by an initial population size of 22,331–26,411 individuals (see Results).

**TABLE 1:** Variable importance (median and confidence interval) for present‐day ensemble modelling of habitat suitability for koalas ( *Phascolarctos cinereus* ) in the Mount Lofty Ranges, South Australia.

Variable	Median	Confidence interval
average rainfall for November (mm)	0.52	[0.49–0.54]
minimum temperature (°C)	0.15	[0.14–0.15]
pH	0.10	[0.10–0.11]
phosphorus	0.05	[0.14–0.05]
likelihood of native vegetation being present in the grid cell (%)	0.03	[0.03–0.03]
solar exposure (MJ m^−2^)	0.02	[0.02–0.03]
Distance to water bodies (m)	0.02	[0.01–0.01]
water vapour pressure (hpa)	0.01	[0.01–0.01]
distance to roads (m)	0	[0–0]
total water index	0	[0–0]
elevation (m)	0	[0–0]

*Note:* Values summarised across all 100 training datasets with the lower and upper limits of the confidence interval calculated as the 2.5^th^ and 97.5^th^ percentiles, respectively.

#### 2.5.2 Projection Scenarios

Baseline (no‐intervention)—To provide a realistic baseline expectation of population trajectory for comparison to the sterilisation interventions, we used the population size estimated from the inhomogeneous Poisson point‐process ensemble distribution model as the founding population size, and expressed all subsequent projections as a proportion of that initial abundance. Because the Mount Lofty Ranges are bounded by habitat that is largely unsuitable for koalas (Sequeira et al. 2014; Whisson and Ashman 2020), making immigration or permanent emigration unlikely, we assumed a closed population structure.

We included a logistic compensatory density‐feedback function by reducing survival as the population approached the carrying capacity of the form:

(6) $$S_{\mathrm{mod}}={\left(a+\mathrm{bN}\right)}^{\left(\frac{-1}{c}\right)}$$

where S_mod_ is the proportion of realised survival (survival modifier) as a function of population size *N*, and the constants *a* = −107, *b* = 0.00216, and *c* = 34.7 (Figure S2). Here, we assumed that survival probability would decline as the population approached a carrying capacity that we assumed was 20% higher than the current population size to allow for additional dispersal into suitable habitats in the Mount Lofty Ranges not currently occupied or at low density.

We also invoked a catastrophic mortality function at a probability of 0.14 generation^−1^ based on Reed et al. (2003) (generation length = 6.75 calculated from the deterministic matrix), where each event would reduce the population by 50% (this percentage stochastically resampled assuming a 5% standard deviation). This function accounts for drought, flood, and fire events, which are integral to Australian ecosystem dynamics (Bowman et al. 2015).

Sterilisation scenarios—We examined two main sterilisation scenarios that represent the two extremes of fertility control options: (i) only adult females sterilised, and (ii) all adult females with back young, plus their daughters sterilised together at capture. For the first scenario, where only adult females are targeted and sterilised, we adjusted the baseline model with incrementing proportional sterilisation of females (expressed as reductions in overall fertility) randomly selected from the adult portion of the age structure. The second scenario is predicated on the notion that capturing adult females with back young and sterilising both mother and daughter would be more efficient and effective than adult‐only sterilisation (Hynes et al. 2019). This is because approximately 47% of koalas surveyed in the Mount Lofty Ranges show signs of chlamydia infection (although based on a sample of only 75 koalas; Fabijan et al. 2019). Using a *b* *eta* *‐*binomial model with a weakly informative prior to account for the small sample size (Harrison 2015), we estimated that up to 11% of infected individuals progress to a diseased state (based on a posterior mean of 5.2% *and* a 95% credible interval of 1.45%–11.11%). Given that 85% of reproductively ‘inactive’ females tested positive for chlamydia in the Mount Lofty Ranges (Fabijan et al. 2019), we might expect up to 4% of reproductive‐age females do not breed as a result of chlamydia disease (i.e., 0.47 × 0.11 × 0.85 = 0.04) and would not require the procedure. Because detecting and capturing individuals is the most time‐consuming and expensive part of the process (not the sterilisation procedure itself), focusing on females proven fertile (those with back young) would optimise the cost–benefit by avoiding the sterilisation of already chlamydia‐infertile females. However, this approach presents ethical challenges related to authorising sterilisation of back young.

#### 2.5.3 Costs

For both models, sterilisation is achieved through subcutaneous hormone implants. These implants release hormones over a prolonged period, interfering with the normal reproductive processes and effectively preventing the animal from breeding. This method presents the main advantages of being non‐surgical and reversible (unlike surgical sterilisation such as spaying or neutering, which are permanent), so that the animal can regain its reproductive capabilities if desired. In the absence of available cost estimates for applying the gestagen implant levonorgestrel, we based our cost estimates on $30 h^−1^ labour cost, 0.83 h koala^−1^ search/capture and $27 cost for each hormone implant (modified from Delean et al. 2013).

## 3 Results

**FIGURE 1:**
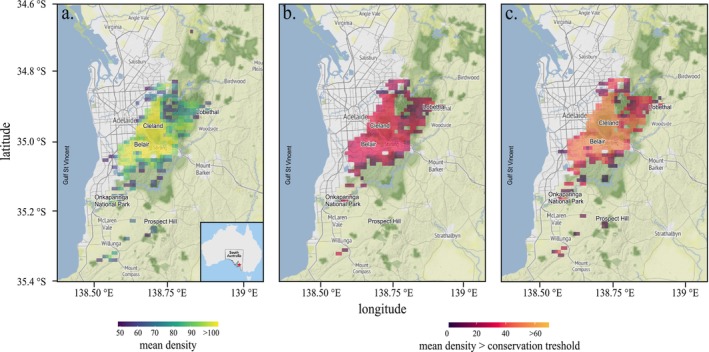
Local koala densities in the Mount Lofty Ranges, South Australia, at a spatial resolution of 1 km × 1 km. (a) Present‐day mean population densities, (b) current simulated mean koala density beyond the density target of 0.7 ha^−1^ (National Parks and Wildlife South Australia 2002; Ramsey et al. 2016), and (c) projections of areas beyond the density target 25 years into the future. Gradients range from dark blue and dark red (low population density) to light green and orange (high population density).

**FIGURE 2:**
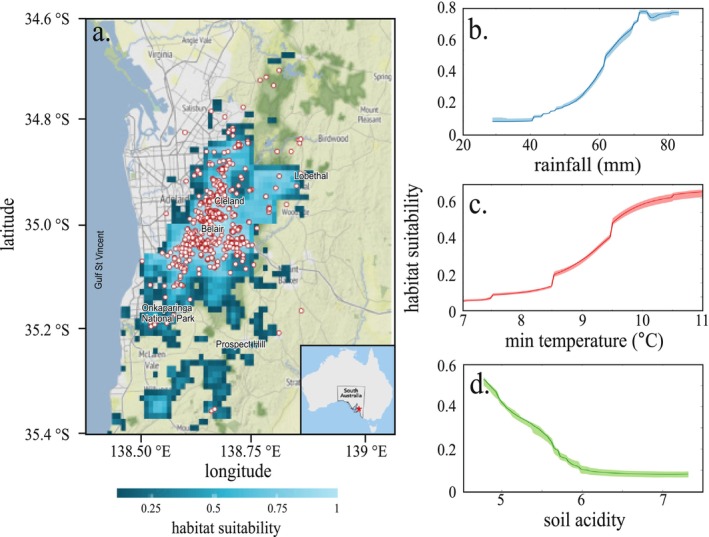
Koala habitat suitability and environmental drivers in the Mount Lofty Ranges. (a) Present day (2003–2018, 16‐year mean) ensemble averaged probability of koala presence across the Mount Lofty Ranges, South Australia, at a spatial resolution of 1 × 1 km. Gradient ranges from dark to light blue, indicating low to high habitat suitability. Ensemble model outputs are based on 9 modelling algorithms (see Methods) for which we calculated a weighted average based on their relative performance. White/red‐circle dots indicate koala presences based on the *Great Koala Count* 1 and 2, grey areas show the urbanised area, and green gradient shows vegetation density from dark to light green. Also shown are the response curves for the three most important predictor variables for koala habitat suitability (Table 1): (b) average rainfall for November (mm, 20‐year average), (c) monthly minimum temperature (°C, 20‐year average), and (d) soil pH (unitless). Envelopes represent the confidence intervals calculated as the 25^th^ and 75^th^ percentiles across 20 different training and evaluation datasets used to generate pseudo‐absences (see Methods). For each predictor tested, we varied values from the minimum to the maximum (100 increments) while holding the other variables constant (at the mean value).

Using the inhomogeneous Poisson point process model, we estimated an average of 106 koalas km^−2^ (95% confidence interval for the average: 104–123 km^−2^) when not constrained by environmentally driven habitat suitability. By rescaling these estimates (i.e., average estimate + confidence intervals) proportionally to habitat suitability, we obtained a total population estimate of 22,761 (22,331–26,411) koalas across the entire study area. The densest areas (up to 123 koalas km^−2^, Figure 1a) are centred around Cleland National Park and Belair National Park in the areas of highest habitat suitability (Figure 2a), and cover an area of approximately 5576 km^2^ (Figure 1a). This density then decreases sharply with distance from this highly suitable core area (~70–80 koalas km^−2^; Figure 1a), especially around the municipality of Lobethal. Some low‐density populations (< 50 koalas km^−2^), are estimated toward the Fleurieu Peninsula in some local areas such as Onkaparinga National Park, Kangarilla, and Prospect Hill that have low habitat suitability for the species (0.5–0.75, Figure 2a).

**FIGURE 3:**
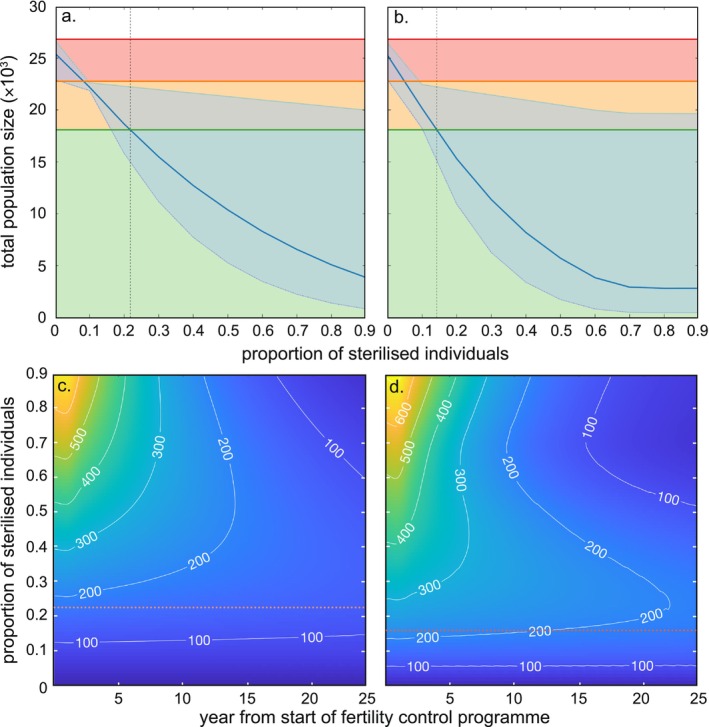
Impact of fertility control on koala population size and its associated cost. (a, b) Projected total population size as a function of the proportion of females sterilised, considering (a) only mature females sterilised, or (b) mature females and their female offspring sterilised. Blue line indicates median values from 10,000 iterations (see Method) and light blue‐shaded areas represent 95% confidence intervals of the simulations calculated from the 95% confidence interval of initial population size (i.e., 22,331–26,411). Also shown are (i) the targeted population size at a threshold of ≤ 0.7 ha^−1^ (horizontal green line and green area), (ii) present‐day reconstructed population size based on an inhomogeneous Poisson point process model combined with a habitat suitability model (horizontal orange line), and (iii) projected unmanaged total population size (horizontal red line). Green area represents all possible population sizes that would meet the density target of 0.7 ha^−1^, and orange and red areas show how much the population size already exceeds this target, or will exceed in the future, if the population is unmanaged. Vertical dotted black line indicates the proportion of females that should be sterilised to meet the density target. (c, d) Projected estimated median yearly costs over time for two sterilisation scenarios: (c) only mature females sterilised, or (d) mature females and their female offspring sterilised. Sterilisation costs (in thousands of AU$) shown as a function of year and the proportion of sterilised individuals; costs are indicated by a colour bar ranging from lowest (dark blue) to highest (yellow). Horizontal dotted red line indicates the proportion of females that should be sterilised to meet the density target. Contours and white values indicate cost isoclines in thousands of AU$.

The ensemble habitat suitability models had high predictive power (AUC = 0.99; Figure 2a). The presence of suitable koala habitat is mostly predicted by rainfall, minimum temperature, and soil acidity (pH) (Table 1). More specifically, the highest habitat suitability was in areas with a high annual rainfall (> 75 mm) (Figure 3b) and warm minimum temperature (> 10°C, Figure 3c), and < 5 pH (Figure 3d). Based on these estimates, applying a density cap of 0.7 koalas ha^−1^ (70 koalas km^−2^) as a threshold for ‘high density’ would indicate > 73% of the current koala distribution predicted to be already beyond this density (Figure 1c) (with a local density ranging from ~8–52 koalas km^−2^, Figure 1b). If unmanaged (i.e., no fertility control), we estimate an increase of ~17%–25% for the population, reaching a total of 26,823 individuals (95% confidence interval: 19,455–32,993; Figure S4 and Table S3) over the next 25 years. Not only would this increase the number of koalas in already high‐density areas, but new areas of high density would appear (e.g., northeast of Mount Compass and Onkaparinga National Park, Lobethal, etc.), leading to > 84% of the suitable population for koalas being categorised as ‘high density’ (Figure 1c).

Fertility control implemented to keep koala density ≤ 0.7 ha^−1^ in the Mount Lofty Ranges, accounting for habitat suitability, requires a total population (i.e., males and females) < 30,194 individuals (green horizontal line, Figure 3a,b). Both fertility‐control scenarios we tested have different impacts on the speed of reduction in total population size (i.e., blue line in Figure 3a,b) depending on the annual effort of sterilisation. They both cause a decrease in the total population as the yearly proportion of sterilised individuals increases (but so does the confidence interval around the total population estimates). However, sterilising *adult females only* slows the rate of population reduction (Figure 2a) compared to sterilising *adult females and female back young* (Figure 3b). This results in a higher yearly proportion of *adult females only* sterilised (~22%, Figure 3a) compared to sterilising *adult females and female back young* (~14%, Figure 3b) to match this conservation target. Sterilising between 5% and 8% of individuals, irrespective of scenario, would merely keep the population constant at its present density, which means there would still be an ‘excess’ of 5231 (95% confidence interval: 4881–8367 individuals; orange area, Figure 3a,b).

The overall cost of sterilising the koala population increases with annual sterilisation rate (Figure 3c,d). Regardless of the scenario, > 15% sterilised individuals year^−1^ leads to the annual costs declining over time. Planning to sterilise 22% of *adult females only* annually to reach the conservation target would be cheaper over time (~AU$34 million in total; Figure 3a) than trying to reach the same conservation target in the *adult females and female back young* scenario (> AU$43 million in total for sterilising 14% of *adult females and female back young*; Figure 3b). This translates into ~AU$1.57 million per percentage point of sterilisation effort for the *adult females only* versus ~AU$3.04 million under the *adult females and female back young* scenario.

**FIGURE 4:**
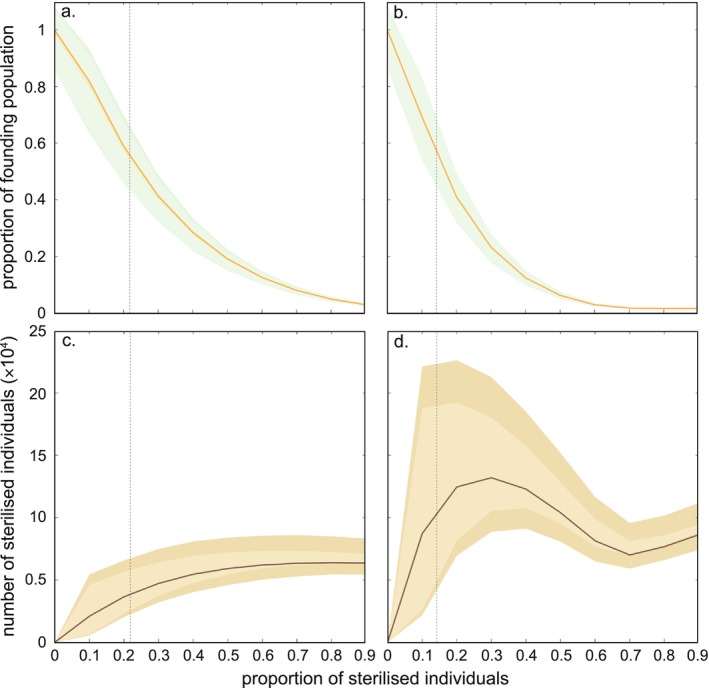
Impact of fertility control on the koala population in the Mount Lofty Ranges, South Australia. (a, b) Projected proportion of the initial koala population (i.e., males and females) and (c, d) number of sterilised koalas as a function of the proportion of females sterilised considering: (a, c) only adult females sterilised, or (b, d) adult females and their female offspring sterilised. Orange (a, b) and brown (c, d) lines indicate median values from 10,000 iterations. Light green‐ (a, b) and light brown‐ (c, d) shaded areas represent 95% confidence intervals from the simulation based on a median initial population size of 22,761 koalas (see Methods). Dark green (a, b) and brown (c, d) envelopes represent 95% confidence intervals calculated from the 95% confidence interval of median initial population size (i.e., 22,331–26,411).

Sterilising both *adult females and female back young* reduced the initial population faster than sterilising *adult females only* (Figure 2a,b), because this scenario increased the proportion of animals sterilised each year. For example, sterilising 50% of *adult females only* (0.5; Figure 4a) would cause a reduction of approximately 80% of the initial population (declining from 1 to 0.2, Figure 4a). In comparison, the same reduction could be achieved by sterilising only 35% of *adult females and female back young* each year (0.35; Figure 4b). To reach the conservation target, this would translate into a decrease of ~44% of the founding population by sterilising 22% of *adult females only* (Figure 4a), while sterilising 14% of *adult females and female back young* would decrease the founding population by ~47% (Figure 4b).

Our simulations projected that the true number of sterilised koalas (males and females) under a sterilised *adult females only* scenario plateaued around 70,000 (Figure 4c), whereas the number of sterilised *adult females and female back young* declined from 1,500,000 to 800,000 at an annual sterilisation rate between 40% and 70%, before reaching 900,000 beyond 70% (Figure 4d). The confidence intervals around the estimates derived for the second scenario (maximum confidence interval width = 2,000,000; Figure 4d) were wider than those for the first scenario (maximum confidence interval width = 400,000; Figure 4c). However, at < 30% of sterilised individuals, targeting *adult females only* produced a slower increase in the number of sterilised individuals compared to targeting *adult females and female back young* (reaching a median of 85,000 versus 153,000 sterilised individuals, respectively; Figure 4c,d). This difference between scenarios has a large effect on the total population sterilised when it comes to meeting the conservation target. The *adult females only scenario* would result in ~38,400 sterilised individuals (Figure 4c), whereas it would reach > 103,000 individuals under the *adult females and female back young* scenario (Figure 4d, and Table S3).

The cost‐effectiveness of fertility control strategies varies depending on the conservation goals set for koalas, particularly the population‐density threshold, so that adjusting these goals affects model outcomes (Table S3). For example, relaxing the density goal to 1 ha^−1^ leads to a 65%–90% decrease in the areas exceeding the target density, reducing the number of sterilised koalas by 73%, and associated costs by 73%–75%. Conversely, a more stringent conservation goal of 0.5 ha^−1^ results in up to a 57%–85% increase in high‐density areas, requiring more control measures to sterilise an extra 25%–40% of individuals and raising costs by 35%–44% (Table S3).

## 4 Discussion

Meaningful non‐lethal conservation strategies (e.g., translocation or fertility control) for managing high‐density species should be grounded in robust spatiotemporal predictions of regional overabundance, careful assessment of implementation costs, and rigorous monitoring of management outcomes (Whisson and Ashman 2020). Using spatially explicit, debiased approaches, we can estimate koala population abundance across the Mount Lofty Ranges to identify areas of current and future high‐density areas, as well as project population trajectories and the relative cost‐effectiveness of management interventions. With an estimated size of 25,733 to 30,435, the population in the Mount Lofty Ranges represents about 10% of Australia's estimated total koala population (Adams‐Hosking et al. 2016). While it is statistically challenging to ground‐truth local population density estimates per km^2^, our estimated range aligns with expert‐elicited estimates (Adams‐Hosking et al. 2016) and reduces previous estimates based on the Great Koala Count by about 29% (Sequeira et al. 2014). The conservation target of maintaining the koala population density below ~0.7 ha^−1^ (National Parks and Wildlife South Australia 2002; Ramsey et al. 2016) already suggests that approximately 73% of the region is at or beyond this density in the areas of highest habitat suitability (Figure 1a,b). In contrast to the declining populations in New South Wales, Australian Capital Territory, and Queensland (McAlpine et al. 2015; Whisson and Ashman 2020), we predict that the population in the Mount Lofty Ranges could theoretically increase by a mean of around 18% (95% confidence interval: −12.9%–24.9%) within 25 years if unmanaged, with sub‐populations within 12% of the current range becoming ‘high‐density’ (Figure 1c). The latter estimate is higher than that proposed by expert elicitation (+3%) (Adams‐Hosking et al. 2016). Although koala populations in other regions of South Australia such as Kangaroo Island and the lower Murray River are expected to suffer population losses, the Mount Lofty Ranges could become a high‐density population centre because of increasing areas with high habitat suitability (Figure 1a–c).

Based on predicted habitat suitability, average November rainfall, minimum temperature, and soil acidity drive the relative abundance of the koala population across the Mount Lofty Ranges (Figure 2). While the relationship between predicted habitat suitability and species abundance varies regionally and by taxon (Dallas and Hastings 2018; Murphy et al. 2006; Rondinini et al. 2011), Australian mammal abundance is generally positively correlated with the outputs of species distribution models (VanDerWal et al. 2009). Such a relationship is important for identifying climate and weather refugia for koalas today and in the future (Kearney et al. 2010; Krockenberger et al. 2012). In the Mount Lofty Ranges, increasing rainfall during the warmer summer months increases habitat suitability (> 70%, Figure 2c) because it mitigates the effects of rising temperatures and drought, and potentially reduces fire risk. While extreme temperatures can increase mortality (Lunney and Hutchings 2012), low minimum temperatures (< 11°C, Figure 2b) challenge koala thermoregulation (Adam et al. 2020). As such, koalas have adapted by selecting specific tree species (Figure 2d) that offer better insulation and moisture (Degabriele and Dawson 1979), such as *Eucalyptus viminalis* and *E. ovata* (Menkhorst 2008) which are preferred in cooler climates for their high moisture and nutrient content (Clifton et al. 2007; Moore and Foley 2005). In addition to their thermal benefits, eucalypts such as manna gum *E. viminalis* also serve as primary food sources (Lee and Martin 1988). Eucalypts are adapted to live in acidic soils (Figure 2d; Evans 1992) because of their symbiotic relationships with ectomycorrhizal fungi (Aggangan et al. 1996; Malajczuk et al. 1975). Although soil pH is not the dominant predictor of habitat suitability (Figure 2b–d), it indirectly affects koala distribution by shaping the availability and condition of important vegetation types (Bui et al. 2017; Hageer et al. 2017).

An unmanaged koala population that increases in density enough to cause damage to vegetation can lead to starvation and reduce the local population's probability of persistence (Todd et al. 2008). Although expensive and controversial, artificial fertility reduction remains a relevant strategy to minimise the likelihood of overabundance leading to catastrophic mortality events (Figure 3a,b). Regulating the koala population via fertility interventions in areas of high habitat suitability (Figure 2) could achieve target densities of ~0.7 ha^−1^. Sterilising both females and their dependent daughters would achieve these conservation goals slightly faster than targeting females only (Figure 3a,b), but the cost difference over the next 25 years would be substantial (i.e., approximately double; Figures 3c,d and 4c,d). Our cost–benefit analysis therefore suggests that focusing on adult female‐only sterilisation is more cost‐effective for fertility control (Figure 3c,d). Adult female‐only sterilisation would have the additional benefit of avoiding the ethical challenges of fertility interventions (i.e., surgical sterilisation, hormonal implant, etc.) in young animals (Australian Government Department of Agriculture, Fisheries and Forestry 2011; Hampton et al. 2015; RSPCA 2024). Regardless of the intervention scenario implemented, the total cost required to achieve acceptable density targets would be < $45 million over the next 25 years, averaging $1.8 million year^−1^ (Figure 3c,d). That amount is < 20% of the Australian Government's investment in wildlife recovery following the 2019–2020 Black Summer bushfires (Quarterly Summary, August 2023). Additionally, even the most expensive fertility reduction scenario is cheaper than the most cost‐effective method to eradicate cats on Kangaroo Island (AU$46.5 million–AU$51.6 million, Venning et al. 2021) and is comparable to the annual cost of deer and pig control and eradication programs in South Australia (~$1.1 million year^−1^; Government of South Australia 2023).

We acknowledge that these results rest on two methodological assumptions: (i) koalas do not exhibit strong social interactions influencing their spatial distribution, and (ii) there is no detection decay during surveys (i.e., all individuals present are observed). The first assumption might be challenged here because, beyond evidence of male competitive exclusion in some parts of Australia (Sharp 1995), the data were collected during the breeding season (November) when male koalas actively seek out females. This can introduce spatial dependencies; although median estimates are likely sound, the associated standard errors and confidence intervals might be too narrow, potentially inflating type I error rates (Dormann et al. 2007). The second assumption can also be violated because koalas can be missed even at close range, especially in areas with dense foliage and tall trees. This detection decay is relevant for citizen‐science data that are prone to uneven observer effort and visibility (Dique et al. 2003). If both assumptions are violated, abundance estimates could be biased low (Royle et al. 2005; Williams et al. 2002) and accompanied by artificially narrow confidence intervals (Dormann et al. 2007). Because we focused on overabundant populations, our results likely reflect conservative estimates and should be interpreted as a minimum baseline. To mitigate these biases, we recommend combining citizen‐science data with complementary survey methods whenever possible (Calenge et al. 2015)—for example, spotlighting with distance sampling or spatially (Cripps et al. 2021) and temporally replicated drone surveys that offer a direct, efficient detection method that holds strong potential to guide on‐ground koala management (Crowther et al. 2021; Witt et al. 2020).

Koala populations face compounded risks from disease and reduced genetic diversity, especially in small, isolated populations (Gates et al. 2025; Schultz et al. 2020; Tarlinton et al. 2021). Chlamydia remains a threat to population viability, mostly because of a lack of male avoidance strategy to mate with infected females, increasing the risk of transmission (Schultz et al. 2020). Although heritable variation in chlamydia susceptibility exists, inbreeding might erode this variation, reducing resilience to future outbreaks (Cristescu et al. 2022). Historical population bottlenecks have further reduced genetic diversity through drift and inbreeding, with documented consequences for fertility, immune function, and adaptability (De Cahsan et al. 2025; Schultz et al. 2020; Tarlinton et al. 2021). In the Mount Lofty Ranges, we estimated approximately up to 4% of reproductive‐age females that might be functionally sterile (see Methods), making our estimates slightly conservative. While we did not explicitly model the effects of disease or genetic factors, future implementation of genetic‐rescue strategies could benefit from predictive models that integrate demographic and genetic feedback to guide management and minimise extinction risk (Beaman et al. 2025).

## 5 Conclusion

Managing threatened species that reach high enough densities to cause vegetation damage in parts of their range is particularly challenging when the species is highly valued by the public. South Australia's koala population exemplifies this conflict, where cultural significance and public sympathy clash with ecological concerns like habitat degradation, especially in the Mount Lofty Ranges. Although koalas have adapted to local environmental conditions, unchecked population growth could lead to vegetation damage, eliciting food shortages and potentially localised die‐offs, suffering, and negative implications for many other forest‐dependent species. We show that despite logistical challenges, spatially targeted fertility control is cost‐effective. In addition to cost, ethical considerations and long‐term conservation goals (such as population‐density thresholds) also play an important role in deciding whether intervention is necessary and socially acceptable.

## Author Contributions

**Frédérik Saltré:** conceptualization (lead), data curation (lead), formal analysis (lead), investigation (lead), methodology (lead), project administration (lead), resources (lead), software (lead), validation (lead), visualization (lead), writing – original draft (lead), writing – review and editing (lead). **Katharina J. Peters:** formal analysis (supporting), investigation (supporting), visualization (equal), writing – original draft (equal), writing – review and editing (supporting). **Daniel J. Rogers:** resources (supporting), writing – original draft (supporting), writing – review and editing (supporting). **Joël Chadoeuf:** formal analysis (equal), methodology (equal), software (equal), writing – original draft (supporting), writing – review and editing (supporting). **Vera Weisbecker:** investigation (supporting), writing – original draft (supporting), writing – review and editing (supporting). **Corey J. A. Bradshaw:** conceptualization (supporting), formal analysis (supporting), funding acquisition (lead), investigation (supporting), methodology (equal), resources (supporting), software (equal), visualization (supporting), writing – original draft (supporting), writing – review and editing (supporting).

## Conflicts of Interest

The authors declare no conflicts of interest.

## Supporting information


**Table S1:** Data (i.e., longitude, latitude, sighting date) from the *Great Koala Count* 1 and 2 used to build the inhomogeneous point process model and the ensemble species distribution model. All personal information of submitters (*“Person_Nam”* column) with randomly generated codes (one unique code *per submitter*).
**Table S2:** Input data on survival and fertility of koalas ( *Phascolarctos cinereus* ) for the demographic model collected from published sources. QLD = Queensland; NSW = New South Wales.
**Table S3:** Sensitivity of sterilisation demographic model (i.e., population densities, sterilisation scenarios and associated costs) to the conservation management density target (ha^−1^).
**Figure S1:** Environmental variables used as predictors to build our species distribution models: (a.) distance to roads (m), (b.) distance to water bodies (m), (c.) water vapour pressure (hPa), (d.) monthly minimum temperature (°C), (e.) average rainfall for November (mm), (f.) solar exposure (MJ m^−2^), (g.) elevation (m), (h.) total water index, (i.) percentage native vegetation cover (%), (j.) soil acidity (pH CaCl_2_, unitless) and (k.) phosphorus content (% of fine soil mass). We used 20‐year monthly averages (from 1993 to 2012) of minimum temperature, water vapour pressure, solar exposure (no data for November 2009), and rainfall, from the Australian Government Bureau of Meteorology (bom.gov.au). We extracted soil pH and phosphorus content from the Soil and Landscape Grid of Australia (Malone and Searle 2024; Viscarra Rossel et al. 2014).

## Data Availability

Data and code that support our findings are openly available at github.com/FredSaltre/Koala_MLR and github.com/BEAM‐NguraNadamari/Koala_MLR.
