# Supplementary material for: Balancing High Densities and Conservation Targets to Optimise Koala Management Strategies
Source: Ecol Evol. 2026 Jan 12;16(1):e72470. doi: 10.1002/ece3.72470 (PMC12796514; doi:10.1002/ece3.72470)
Supplement: Supplementary file 1 — Table S1: Data (i.e., longitude, latitude, sighting date) from the Great Koala Count 1 and 2 used to build the inhomogeneous point process model and the ensemble species distribution model. All personal information of submitters (“Person_Nam” column) with randomly generated codes (one unique code per submitter). Table S2: Input data on survival and fertility of koalas ( Phascolarctos cinereus ) for the demographic model collected from published sources. QLD = Queensland; NSW = New South Wales. Table S3: Sensitivity of sterilisation demographic model (i.e., population densities, sterilisation scenarios and associated costs) to the conservation management density target (ha−1). Figure S1: Environmental variables used as predictors to build our species distribution models: (a.) distance to roads (m), (b.) distance to water bodies (m), (c.) water vapour pressure (hPa), (d.) monthly minimum temperature (°C), (e.) average rainfall for November (mm), (f.) solar exposure (MJ m−2), (g.) elevation (m), (h.) total water index, (i.) percentage native vegetation cover (%), (j.) soil acidity (pH CaCl2, unitless) and (k.) phosphorus content (% of fine soil mass). We used 20‐year monthly averages (from 1993 to 2012) of minimum temperature, water vapour pressure, solar exposure (no data for November 2009), and rainfall, from the Australian Government Bureau of Meteorology (bom.gov.au). We extracted soil pH and phosphorus content from the Soil and Landscape Grid of Australia (Malone and Searle 2024; Viscarra Rossel et al. 2014). [file ECE3-16-e72470-s001.zip › ece372470-sup-0001-Supinfo.docx]

**Balancing high densities and conservation targets to optimise koala management strategies**

Frédérik Saltré, Katharina J. Peters, Daniel J. Roger, Joël Chadoeuf, Vera Weisbecker, Corey J. A. Bradshaw

**Supplementary Information**

**Table S1 (additional file)**. Data (i.e., longitude, latitude, sighting date) from the *Great koala count* 1 and 2 used to build the inhomogeneous point process model and the ensemble species distribution model. All personal information of submitters (*“Person_Nam”* column) with randomly generated codes (one unique code per submitter).

**Table S2**. Input data on survival and fertility of koalas (*Phascolarctos* *cinereus*) for the demographic model collected from published sources. QLD = Queensland; NSW = New South Wales

| **sex** | **age** | **State** | **population** | **survival** | **SE** | **fertility** | **SD** | **Reference** |
| --- | --- | --- | --- | --- | --- | --- | --- | --- |
| female | 0-1 | QLD | Koala Coast | 0.13 | 0.08/0.18 | N/A | N/A | Rhodes et al. 2011 |
| female | 1-2 | QLD | Koala Coast | 0.06 | 0.08/0.36 | 0.08 | 0.02/0.19 | Rhodes et al. 2011 |
| female | 2-3 | QLD | Koala Coast | 0.09 | 0.16/0.46 | 0.47 | 0.3/0.65 | Rhodes et al. 2011 |
| female | 3+ | QLD | Koala Coast | 0.08 | 0.18/0.34 | 0.71 | 0.65/0.75 | Rhodes et al. 2011 |
| male | 0-1 | QLD | Koala Coast | 0.17 | 0.12/0.22 |  |  | Rhodes et al. 2011 |
| male | 1-2 | QLD | Koala Coast | 0.05 | 0.09/0.44 |  |  | Rhodes et al. 2011 |
| male | 2-3 | QLD | Koala Coast | 0.13 | 0.38/0.89 |  |  | Rhodes et al. 2011 |
| male | 3+ | QLD | Koala Coast | 0.07 | 0.23/0.46 |  |  | Rhodes et al. 2011 |
| female | 0-1 | QLD | Oakey | 0.33 | 0.033 |  |  | Penn et al. 2000 |
| female | 1-2 | QLD | Oakey | 0.17 | 0.017 |  |  | Penn et al. 2000 |
| female | 2-3 | QLD | Oakey | NA | NA |  |  | Penn et al. 2000 |
| female | 3+ | QLD | Oakey | 0.09 | 0.009 |  |  | Penn et al. 2000 |
| male | 0-1 | QLD | Oakey | 0.2 | 0.02 |  |  | Penn et al. 2000 |
| male | 1-2 | QLD | Oakey | 0.23 | 0.023 |  |  | Penn et al. 2000 |
| male | 2-3 | QLD | Oakey | 0.23 | 0.023 |  |  | Penn et al. 2000 |
| male | 3+ | QLD | Oakey | 0.26 | 0.026 |  |  | Penn et al. 2000 |
| female | 0-1 | QLD | Springsure | 0.3 | 0.003 |  |  | Penn et al. 2000 |
| female | 1-2 | QLD | Springsure | 0.16 | 0.002 |  |  | Penn et al. 2000 |
| female | 2-3 | QLD | Springsure | NA | NA |  |  | Penn et al. 2000 |
| female | 3+ | QLD | Springsure | 0.08 | 0.008 |  |  | Penn et al. 2000 |
| male | 0-1 | QLD | Springsure | 0.2 | 0.02 |  |  | Penn et al. 2000 |
| male | 1-2 | QLD | Springsure | 0.23 | 0.023 |  |  | Penn et al. 2000 |
| male | 2-3 | QLD | Springsure | 0.23 | 0.023 |  |  | Penn et al. 2000 |
| male | 3+ | QLD | Springsure | 0.26 | 0.026 |  |  | Penn et al. 2000 |
| female | 0-1 | QLD | Koala Coast | 0.11 |  |  |  | Dique et al. 2003 |
| female | 1-2 | QLD | Koala Coast | 0.06 |  |  |  | Dique et al. 2003 |
| female | 2-3 | QLD | Koala Coast | 0.25 |  |  |  | Dique et al. 2003 |
| female | 3+ | QLD | Koala Coast | NA |  |  |  | Dique et al. 2003 |
| male | 0-1 | QLD | Koala Coast | 0.17 |  |  |  | Dique et al. 2003 |
| male | 1-2 | QLD | Koala Coast | 0 |  |  |  | Dique et al. 2003 |
| male | 2-3 | QLD | Koala Coast | 0 |  |  |  | Dique et al. 2003 |
| male | 3+ | QLD | Koala Coast | NA |  |  |  | Dique et al. 2003 |
| female | 0-1 | NSW | Port Stephens | 0.4 | 0.04 |  |  | Lunney et al. 2007 |
| female | 1-2 | NSW | Port Stephens | 0.4 | 0.04 |  |  | Lunney et al. 2007 |
| female | 2-3 | NSW | Port Stephens | NA | NA |  |  | Lunney et al. 2007 |
| female | 3+ | NSW | Port Stephens | 0.23 | 0.023 |  |  | Lunney et al. 2007 |
| male | 0-1 | NSW | Port Stephens | 0.4 | 0.04 |  |  | Lunney et al. 2007 |
| male | 1-2 | NSW | Port Stephens | 0.4 | 0.04 |  |  | Lunney et al. 2007 |
| male | 2-3 | NSW | Port Stephens | 0.4 | 0.04 |  |  | Lunney et al. 2007 |
| male | 3+ | NSW | Port Stephens | 0.39 | 0.039 |  |  | Lunney et al. 2007 |

**Table S3** – Sensitivity of sterilization demographic model (i.e., population densities, sterilization scenarios and associated costs) to the conservation management density target (ha^-1^)

|  | | population density target (ha^-1^) | | | |
| --- | --- | --- | --- | --- | --- |
|  | 0.7 | | 0.5 | 1 |  |
| present-day mean population densities > density target | 5,231 | | 9,666 (+85%)  [9,243 – 13,311] | 524 (-90%)  [317 – 2766] |  |
|  | | | | | |
| *no sterilization* | | | | | |
| projected future mean koala population size | 26,823  [19,455 – 32,993] | | - | - |  |
| projected mean koala > density target | 8737  [8,073 – 12,341] | | 13,723 (+57%)  [12,983 – 17,549] | 3055 (-65%)  [2541 – 5,969] |  |
|  | | | | | |
| *only mature females sterilized* | | | | | |
| targeted proportion of sterilized females | 0.22 | | 0.39 (+77%) | 0.05 (-77%) |  |
| proportion of founding population | 0.56 | | 0.30 (-46%) | 0.91 (+63%) |  |
| number of sterilized koalas | 38,400 | | 53,635 (+40%) | 10,478 (-73%) |  |
| Total sterilisation cost (AU$) | 34,235,906 | | 49,379,604 (+44%) | 9,136,981 (-73%) |  |
|  |  | |  |  |  |
| *mature females and their female offspring sterilized* | | | | | |
| targeted proportion of sterilized females | 0.14 | | 0.26 (+86%) | 0.03 (-79%) |  |
| proportion of founding population | 0.57 | | 0.31 (-46%) | 0.91 (+60%) |  |
| number of sterilized koalas | 103,100 | | 128,776 (+25%) | 27,500 (-73%) |  |
| Total sterilisation cost (AU$) | 43,251,626 | | 58,210,222 (+35%) | 10,811,775 (-75%) |  |
|  |  | |  |  |  |

**Fig. S1.** Environmental variables used as predictors to build our species distribution models: (a.) distance to roads (m), (b.) distance to water bodies (m), (c.) water vapour pressure (hPa), (d.) monthly minimum temperature (°C), (e.) average rainfall for November (mm), (f.) solar exposure (MJ m^-2^), (g.) elevation (m), (h.) total water index, (i.) percentage native vegetation cover (%), (j.) soil acidity (pH CaCl_2_, unitless) and (k.) phosphorus content (% of fine soil mass). We used 20-year monthly averages (from 1993 to 2012) of minimum temperature, water vapour pressure, solar exposure (no data for November 2009), and rainfall, from the Australian Government Bureau of Meteorology (bom.gov.au). We extracted soil pH and phosphorus content from the Soil and Landscape Grid of Australia (Malone & Searle, 2024; Viscarra Rossel et al., 2014).

**
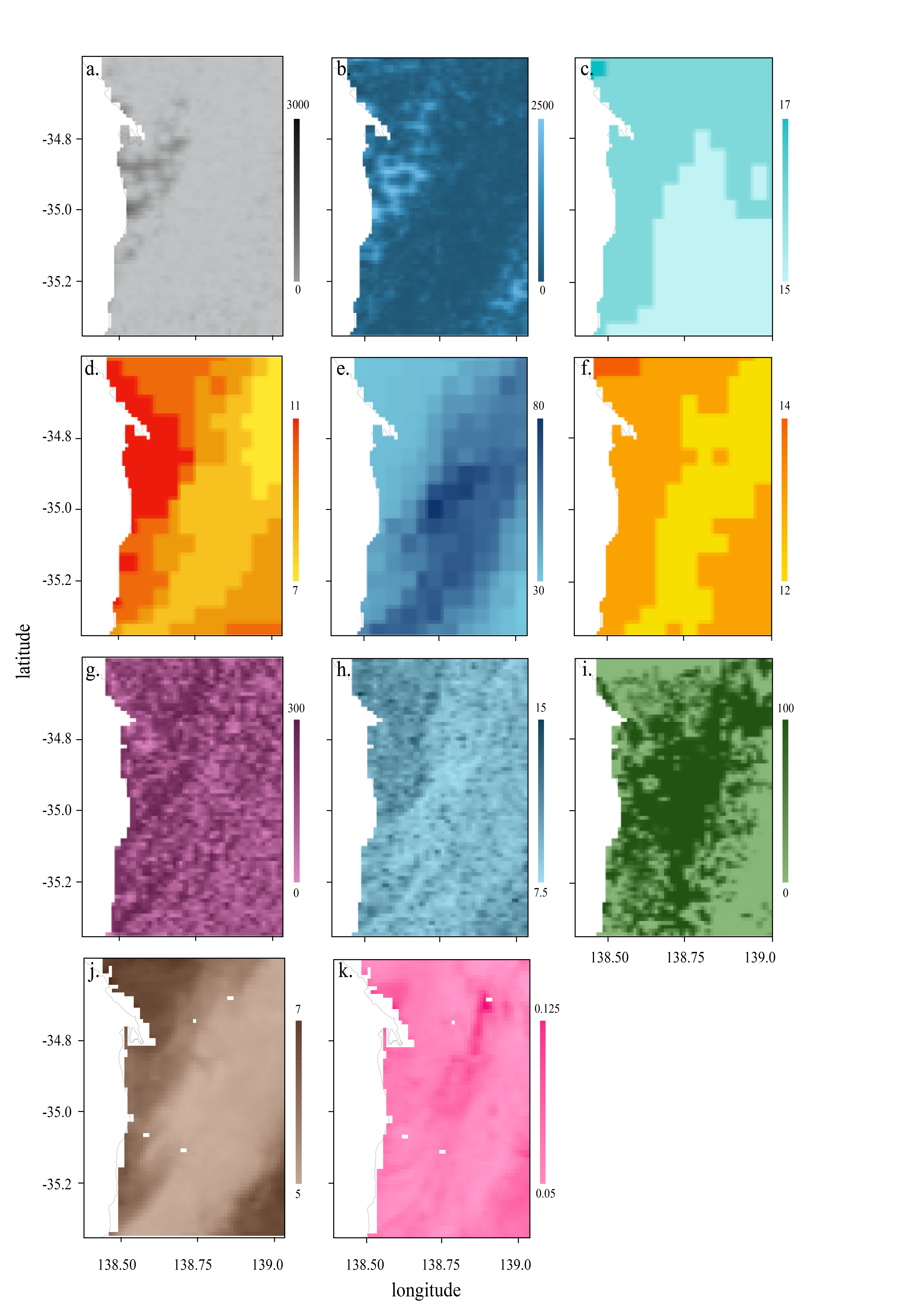
**

**Fig. S2.** Fertility and survival data


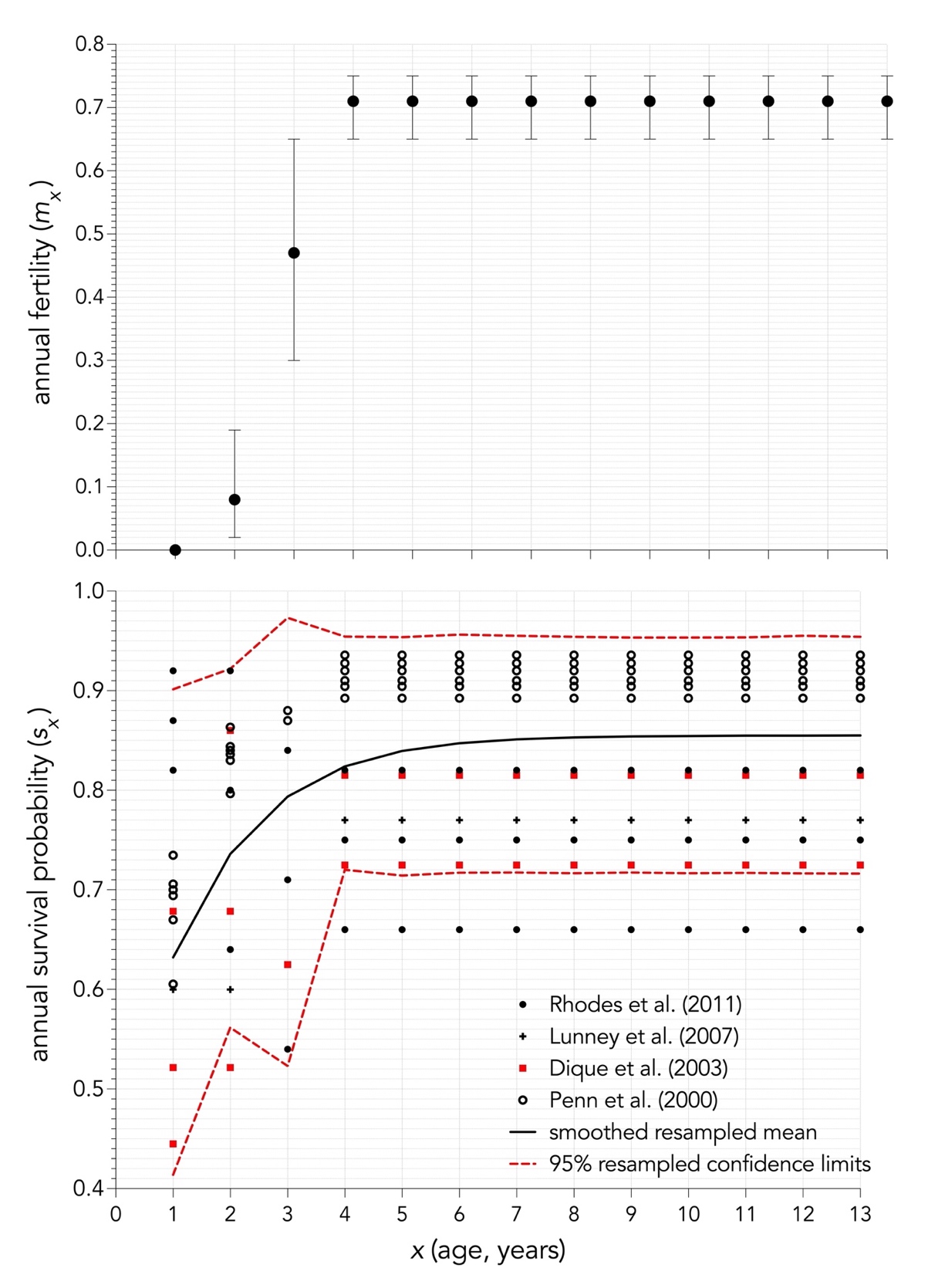


**Fig. S3**. Median true skill statistic (TSS) and median area under the receiver operating curve (AUC) for training and test datasets. For each model, the confidence intervals represent the 2.5^th^ and 97.5^th^ percentile calculated across all 20 different training and evaluation datasets. Ensemble averaged model (black dot) runs using training and test datasets obtained TSS scores of > 0.8 for all nine algorithms: artificial neural networks (ANN), generalized additive models (GAM), generalized linear models (GLM), boosted regression trees (GBM), flexible discriminant analysis (FDA), multivariate adaptive regression splines (MARS), maximum entropy (MAXENT), random forest (RF), and species-range envelopes (SRE).


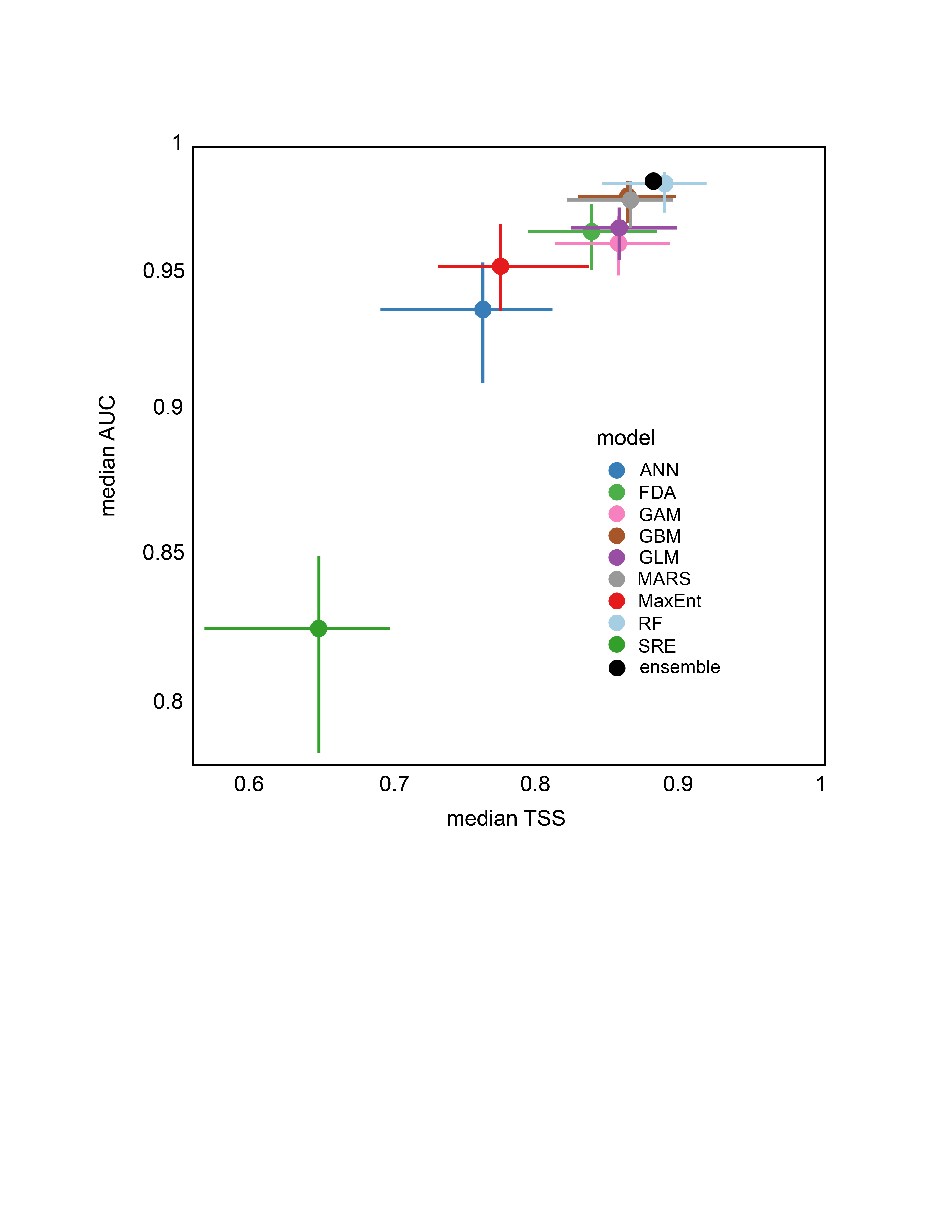


**Fig. S4.** Expected population size (*N*) assuming no-intervention scenario projected over the next 25 years. The solid blue line indicates the median values from 10,000 iterations and the envelope represents the 95% confidence interval calculated from the 95% confidence interval of median initial population size (i.e., 22,331–26,411).


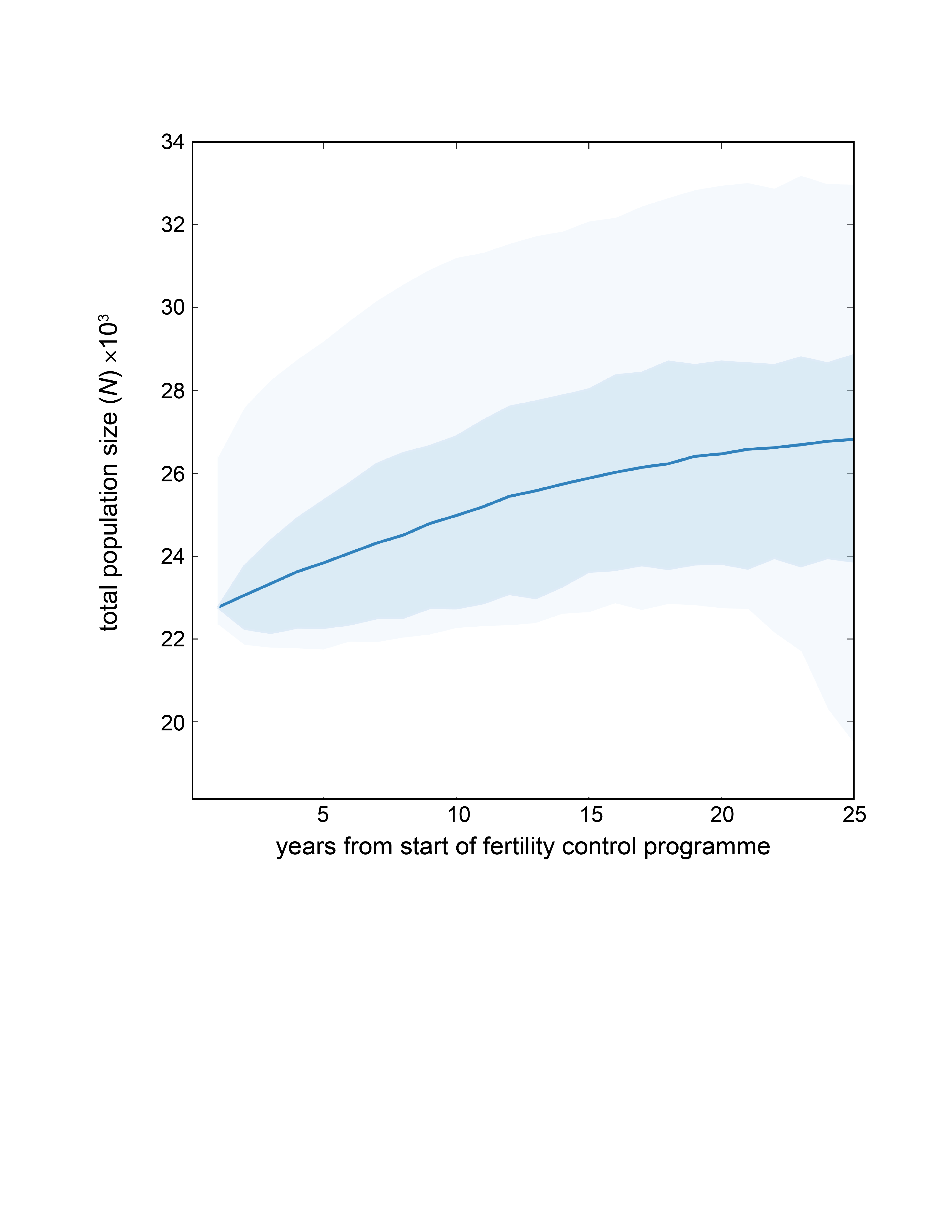


**References**

Dique, D. S., Villiers, D. L. d., & Preece, H. J. (2003). Evaluation of line-transect sampling for estimating koala abundance in the Pine Rivers Shire, south-east Queensland. *Wildlife Research, 30*(2), 127-133. doi:<https://doi.org/10.1071/WR02042>

Lunney, D., Gresser, S., O'Neill, L. E., Matthews, A., & Rhodes, J. (2007). The impact of fire and dogs on Koalas at Port Stephens, New South Wales, using population viability analysis. *Pacific Conservation Biology, 13*(3), 189-201. doi:<https://doi.org/10.1071/PC070189>

Malone, B., & Searle, R. (2024). *Soil and Landscape Grid National Soil Attribute Maps - Total Phosphorus (3" resolution)*.

Penn, A. M., Sherwin, W. B., Gordon, G., Lunney, D., Melzer, A., & Lacy, R. C. (2000). Demographic Forecasting in Koala Conservation. *Conservation Biology, 14*(3), 629-638. doi:<https://doi.org/10.1046/j.1523-1739.2000.99385.x>

Rhodes, J. R., Ng, C. F., de Villiers, D. L., Preece, H. J., McAlpine, C. A., & Possingham, H. P. (2011). Using integrated population modelling to quantify the implications of multiple threatening processes for a rapidly declining population. *Biological Conservation, 144*(3), 1081-1088. doi:<https://doi.org/10.1016/j.biocon.2010.12.027>

Viscarra Rossel, R., Chen, C., Grundy, M., Searle, R., Clifford, D., Odgers, N., . . . Kidd, D. (2014). *Soil and Landscape Grid National Soil Attribute Maps - pH - CaCl2 (3" resolution)*.
